# Reviewed article: Carvalho AF, Guaranha DDFK, Marmett B, Reis JM, Rosa BS, Souza CLE, Dalcin TC, Amantea SL, Machado FV. A multicenter study on the mental health of Brazilian adolescent mothers, 2024. Epidemiol Serv Saude. 2025:34;e20240226.

**DOI:** 10.1590/S2237-96222025v34e20240226.c

**Published:** 2025-09-08

**Authors:** Maria Cristina Mazzaia

**Affiliations:** 1 UNIFESP, Enfermagem Clínica e Cirúrgica

**Table:** 

Rating	Excellent	Good	Average	Below average	Poor
Interest		✓			
Quality			✓		
Originality		✓			
Overall		✓			

**Table:** 

Questionnaire	Yes	No	Not applicable
Does the manuscript contain new and significant information to justify publication?	✓		
Does the Abstract (Summary) clearly and accurately describe the content of the article?		✓	
Is the problem significant and concisely stated?	✓		
Are the methods described comprehensively?	✓		
Are the interpretations and conclusions justified by the results?		✓	
Is adequate reference made to other work in the field?		✓	

## Manuscript Structure

Length of article is: Adequate

Number of tables is: Adequate

Number of figures is: Adequate

Please state any conflict(s) of interest that you have in relation to the review of this paper (state “none” if this is not applicable): None

## Comments

Parabenizo os autores pela iniciativa, tema relevante que trouxe muitos dados que podem contribuir com a individualização dos cuidados para este momento delicado do desenvolvimento, agravado pela situação da gestação.

No resumo destaco que a conclusão necessita resumir e destacar os principais achados.

O tema é introduzido de forma a levar o leitor para o objetivo do estudo e método, que está apresentado de forma descritiva evidenciando todos os procedimentos utilizados para chegar aos resultados, no entanto, o texto carece de adequações ortográficas e gramaticais tornando os procedimentos mais claros ao leitor. Da forma como está apresentado dificulta esta compreensão .

Os resultados são apresentados de forma descritiva e em tabelas e figuras que contribuem para a compreensão dos resultados obtidos. O texto dos resultados também necessita de revisão ortográficas e gramaticais, o que tornará a leitura mais compreensível.

A relação dos resultados entre si e também com a literatura deve ser contemplada na discussõe, no entanto, o texto da discussão reapresenta os resultados, inclusive sendo utilizado todo um parágrafo para tal, sem explorar estas relações. Esta parte do artigo deve valorizar ainda mais os resultados obtidos. Também a discussão carece de revisão ortográfica e gramatical.

Quanto às referências, observo somente 12 referências publicadas nos últimos 5 anos sendo que 4 são documentos técnicos. A busca por artigos mais recentes pode contribuir para a discussão dos resultados obtidos.

Anexo o arquivo com apontamentos realizado durante a leitura, inclusive com sugestões de correções ortográficas e gramaticais.

Atenciosamente,

